# The Effect of Seasonal Floods on Health: Analysis of Six Years of National Health Data and Flood Maps

**DOI:** 10.3390/ijerph15040665

**Published:** 2018-04-03

**Authors:** Dell D. Saulnier, Claudia Hanson, Por Ir, Helle Mölsted Alvesson, Johan von Schreeb

**Affiliations:** 1 Department of Public Health Sciences, Karolinska Institute, 17177 Stockholm, Sweden; claudia.hanson@ki.se (C.H.); helle.molsted-alvesson@ki.se (H.M.A.); johan.von.schreeb@ki.se (J.v.S.); 2 National Institute of Public Health, Ministry of Health, Phnom Penh, Cambodia; ipor@niph.org.kh

**Keywords:** floods, diarrhea, acute respiratory infections, skin infections, injuries, healthcare facilities, disasters

## Abstract

There is limited knowledge on the effect of seasonal flooding on health over time. We quantified the short- and long-term effects of floods on selected health indicators at public healthcare facilities in 11 districts in Cambodia, a flood-prone setting. Counts of inpatient discharge diagnoses and outpatient consultations for diarrhea, acute respiratory infections, skin infections, injuries, noncommunicable diseases and vector-borne diseases were retrieved from public healthcare facilities for each month between January 2008 and December 2013. Flood water was mapped by month, in square kilometers, from satellite data. Poisson regression models with three lag months were constructed for the health problems in each district, controlled for seasonality and long-term trends. During times of flooding and three months after, there were small to moderate increases in visits to healthcare facilities for skin infections, acute respiratory infections, and diarrhea, while no association was seen at one to two months. The associations were small to moderate, and a few of our results were significant. We observed increases in care seeking for diarrhea, skin infections, and acute respiratory infections following floods, but the associations are uncertain. Additional research on previous exposure to flooding, using community- and facility-based data, would help identify expected health risks after floods in flood-prone settings.

## 1. Introduction

Floods affect about 215 million people per year, 95% of whom live in Asia [1]. Systematic reviews have reported mortality, injuries, poisonings, vector- and water-borne diseases, worsening chronic diseases, and malnutrition as health outcomes after floods. Drowning and injuries are considered a direct consequence of contact with flood waters and debris. Landmines can also resurface during floods, increasing the risk for injury. The risk of infectious diarrhea, respiratory and skin infections, and vector-borne diseases has been linked to indirect outcomes of floods, such as contaminated water, greater exposure to vectors, displacement, and overcrowding. Increases in stress and decreased access to healthcare services are considered potential risk factors after floods for the exacerbation or onset of some noncommunicable diseases (NCDs) [2,3,4,5].

Most evidence on the relationship between floods and health is on the immediate effects and comes from large-scale, sudden-onset disasters [2,6,7]. Floods are seasonal and regular in Southeast Asia, caused by monsoon rains and the Mekong River flood pulse [8], although these floods can be unexpectedly severe, such as the flood disasters that occurred in Thailand, Cambodia, Laos, and Vietnam during 2011 [9]. Annual floods are an opportunity to systematically study the effect of floods on health over short and long time periods, in a predictable setting. However, less is known about how these seasonal floods affect health. The aim of the study was to quantify the short- and long-term effects of floods on selected health problems at public healthcare facilities between 2008 and 2013 in 11 districts in Cambodia, a flood-prone setting.

## 2. Materials and Methods

The study was conceived as a six-year time series analysis that would capture the effect of the flood water on care seeking for diarrhea, acute respiratory infections, skin infections, vector-borne diseases, injuries, and NCDs (diabetes and heart disease). We hypothesized that there would be an association between the number of square kilometers of flood water and the number of visits for these health problems each month to public healthcare facilities, and that the rates would increase in the months following the onset of flooding. Satellite imagery was used to map the exposure, which was flood water, and health problem outcomes were measured using healthcare facility data.

Eleven operational districts (referred to as districts for the remainder of the text) in Prey Veng and Kampot provinces in southern Cambodia were included. The districts were chosen because of their previous flood exposure. Prey Veng province is located in the Mekong River floodplain and floods seasonally. Kampot province is located on the coast and floods occasionally. In late 2011 and 2013, both provinces were affected by flood disasters; over 1.2 million people were affected in Cambodia during the disasters [10,11]. Prey Veng province had a population of 947,357 in 2008, and Kampot had a population of 627,884. Each province contains one provincial hospital, and multiple districts with a district referral hospital and several health centers in each (Table 1).

### 2.1. Data

The data came from two main sources: satellite imagery from the National Aeronautics and Space Administration’s (NASA) Moderate Resolution Imaging Spectroradiometer (MODIS) sensors to quantify flood water, and health data from Cambodia’s Health Management Information System (HMIS), which collects data from all healthcare facilities in the public sector [12].

We defined floods as the presence of water on land that is not normally covered by water. To measure the square kilometers of flood water in each district each month, flood water maps from the MODIS Near Real-Time Global Flood Mapping project were used [13]. The aim of the project was to produce global, near real-time flood maps. The project used data from the MODIS sensors on NASA’s Terra and Aqua satellites to produce daily water maps of the earth’s surface at 250-m spatial resolution. Water and cloud cover were classified using ratios from spectral Bands 1 and 2, and a threshold value from Band 7. The project used the NASA MOD44W product (USGS Earth Resources Observation and Science Center, Sioux Falls, South Dakota, USA), a land-water mask compiled from historical MODIS data, as reference water (i.e., locations that are normally covered by water).

Three-day composite flood water maps from the Global Flood Mapping project were acquired for each day between 1 January 2008 and 31 December 2013. In the composite image product, images from the three previous, consecutive days were combined into a single image to minimize insufficient data from cloud cover. If water was observed in a pixel outside of the reference water boundaries over all three days, the pixel was marked as flood water. The composite image approach had previously performed well in detecting flood events [14].

**Figure 1:**
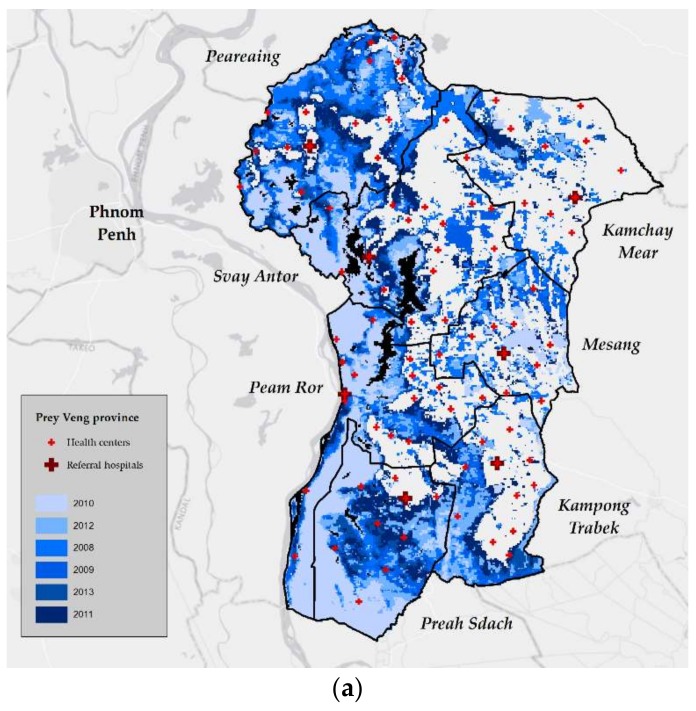

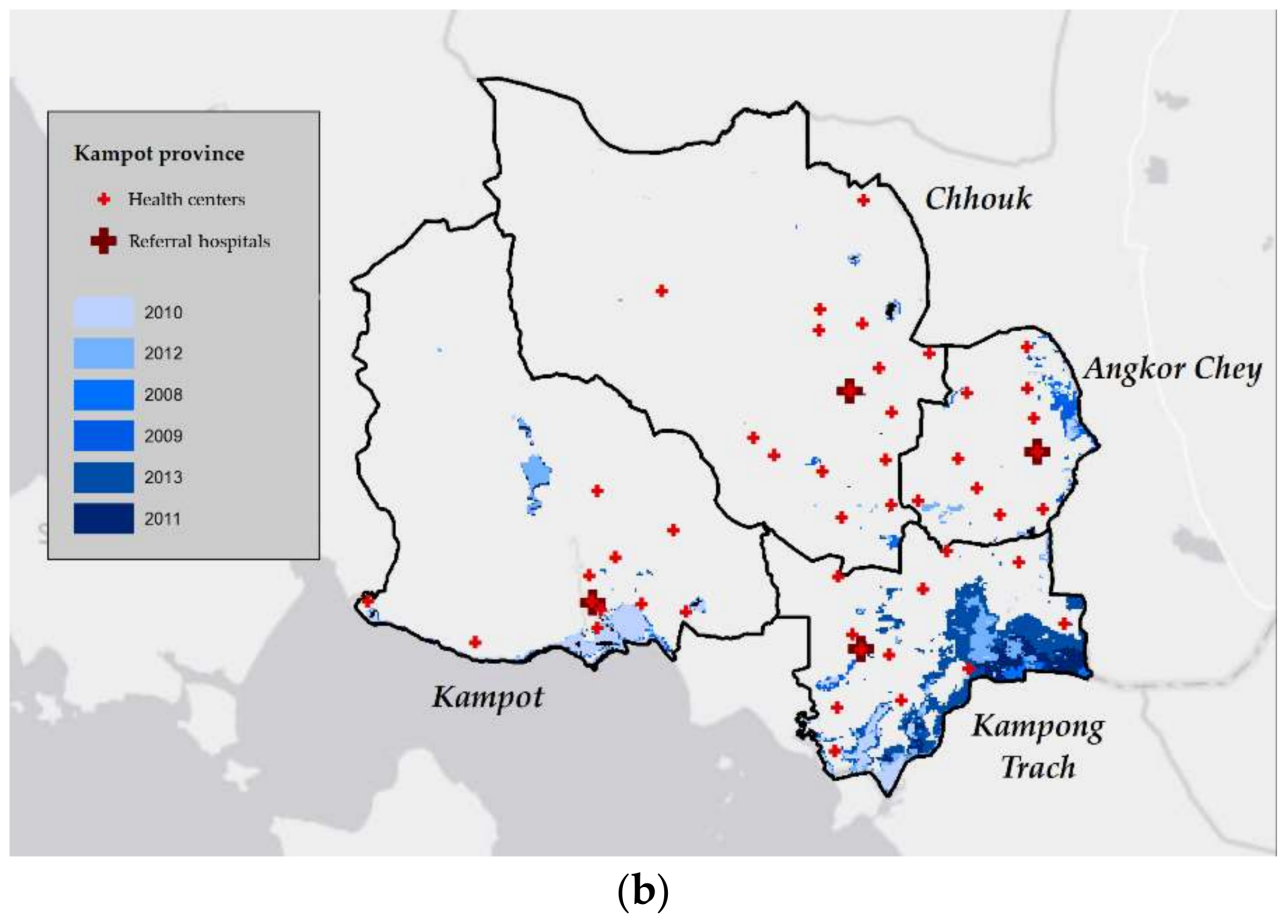
The total extent of flood water per year, and the location of healthcare facilities in 2010, in (**a**) Prey Veng province and (**b**) Kampot province.

To estimate the number of square kilometers of flood water each month in the districts, the composite images were divided by date into the 72 months of the study period. The images were mosaicked into single raster images for each month in ArcMap (Esri ArcGIS Desktop, version 10.5.1.7333, Redlands, CA, USA). The maximum pixel value was used as the mosaic operator, since flood water pixels were marked with the highest value. An administrative map of district boundaries from the Cambodian Ministry of Health was overlaid on the flood maps (Figure 1). Zonal statistics were used to compute the total number of flood water pixels in the mosaicked images, and the number of flood water pixels was converted into the number of square kilometers flooded.

We identified six types of health problems that have previously been reported as directly or indirectly linked to floods, from a review of the literature: diarrhea, acute respiratory infections, skin infections, vector-borne diseases, injuries, and NCDs [2,3,4]. Inpatient and outpatient information was extracted from the HMIS system for all public health centers and hospitals. Monthly summaries of curative care, among other services, were collected by healthcare facility staff from facility logbooks. Paper reports were either sent to the district health departments for data entry or were entered directly by staff into the online database. For curative care, healthcare facilities reported the number of inpatient discharges and outpatient consultations seen each month, by diagnosis or reason for consultation. The HMIS was highly consistent and reporting was judged to be between 99% and 100% complete [15,16].

**Figure 2:**
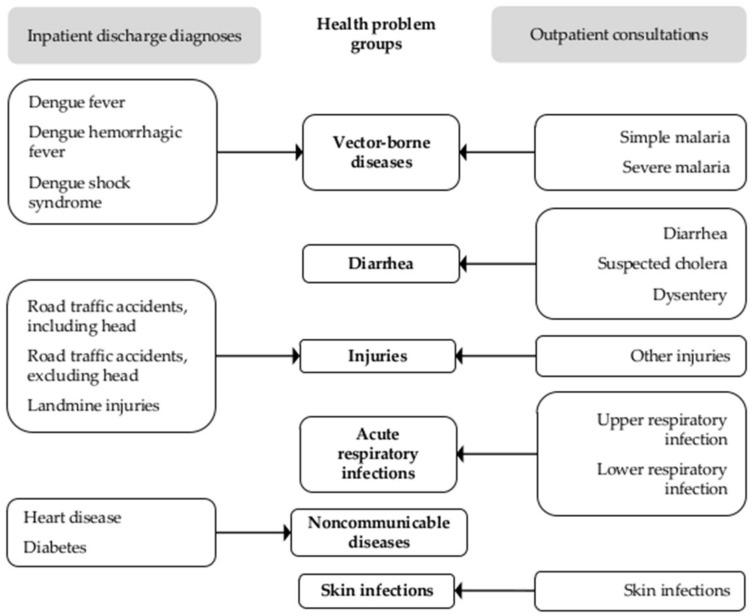
Inpatient discharge diagnosis and outpatient consultation data included in the health problem groups.

We retrieved monthly counts for twenty diagnoses and consultations and combined them into our six health problem groups (Figure 2). Outpatient consultations captured minor complaints and problems that were routinely treated at health centers. Inpatient diagnoses captured health problems that required advanced care or were not routinely treated at health centers. At the time of the study, cases of heart disease and diabetes were reported to HMIS only from inpatient facilities; outpatient facilities did not report NCD cases to HMIS. The data covered all public health centers and hospitals located in the 11districts for the 72 months between January 2008 and December 2013, and was aggregated by district. Complete data on injuries was available only between June 2009 and December 2013.

Twenty-nine extreme outliers in seven districts were identified in the data and considered to be potential data-entry errors in HMIS after examining the original data. Values for these months were imputed by taking the average of the counts in the month before and month after the extreme value; there were no two consecutive months of outliers.

The National Ethics Committee for Human Research in Cambodia granted ethical approval for this study (reference 088, dated 13 March 2017), and permission to use the HMIS data for secondary data analysis. The Ministry of Health in Cambodia gave access to the HMIS data. HMIS data is routinely collected data for which no patient consent is necessary.

### 2.2. Analysis

We used a time series analysis [17]. Poisson regression models with robust standard errors were constructed for each of the six health problem groups in all 11 districts. One- to three-month exposure delays were included to capture possible short- and long-term delayed associations between floods and the health problems. The models included three distributed lag months for all groups except injuries, where a delayed association was not expected [2,3].

To control for seasonality, a binary indicator variable for season was introduced to the models, defined as either rainy months (May to November) or dry months (December to April). A yearly indicator variable was added to control for long-term trends. Sensitivity analyses were conducted to check the robustness of using yearly time intervals to control for long-term trends, compared to restricted cubic splines and six-month time intervals. Yearly intervals were chosen to retain simplicity while still accurately capturing long-term patterns. The final models were three-month unconstrained distributed lag models, controlling for season and year. Each regression model was run with and without the imputed data. The results from both the imputed and the non-imputed data are presented.

To check if flood water affected access to healthcare facilities, and therefore was influencing the number of consultations and discharges at the healthcare facilities, additional analyses using the number of facility visits and deliveries as proxies for access were performed [18]. The total number of outpatient consultations per month was used as the dependent variable in the three-month distributed lag models, controlling for season and year. The total number of deliveries at healthcare facilities, complicated deliveries (including Caesarean sections) at healthcare facilities, and deliveries at home were used individually as dependent variables in models controlling for season and year, with no lags.

The results were presented as the ratio of the incidence rates (counts per month for each health problem) for an increase of ten square kilometers in flood water, after adjusting for season and long-term trends. Stata 14 was used for analysis. All model estimates are presented in Table S1.

## 3. Results

**Figure 3:**
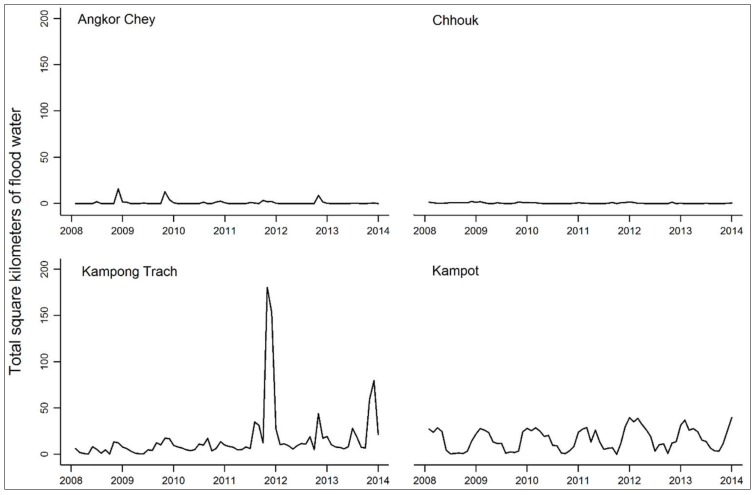
Total number of square kilometers flooded in the districts of Kampot province between January 2008 and December 2013.

**Figure 4:**
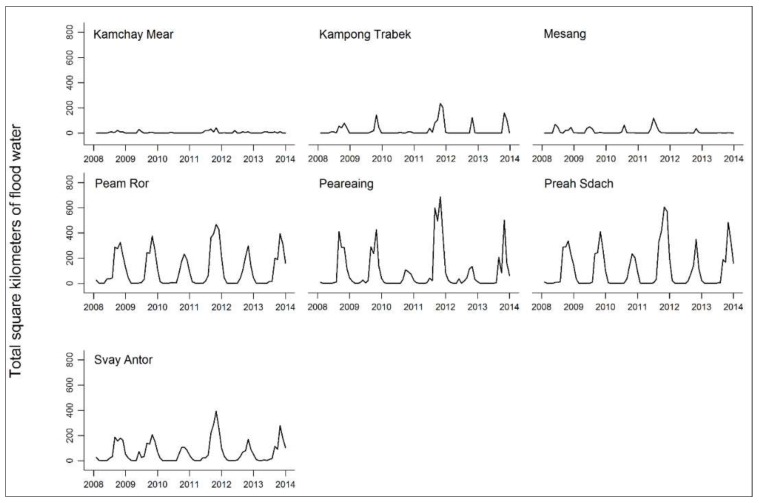
Total number of square kilometers flooded in the districts of Prey Veng province between January 2008 and December 2013.

Between 2008 and 2013, there were 204,186 inpatient discharge diagnoses and 5,544,051 outpatient consultations at public health facilities in the 11 districts. The greatest number of visits were for acute respiratory infections and diarrhea (Table 2). NCDs accounted for 0.04% of visits. Floods followed a clear seasonal pattern in nine of the districts (Figure 3 and Figure 4). High peaks in the number of square kilometers flooded at the end of 2011 relate to the flood disaster that occurred in Cambodia that year. The mean area flooded per month ranged from less than one square kilometer to 106 square kilometers (Table 2).

**Figure 5:**
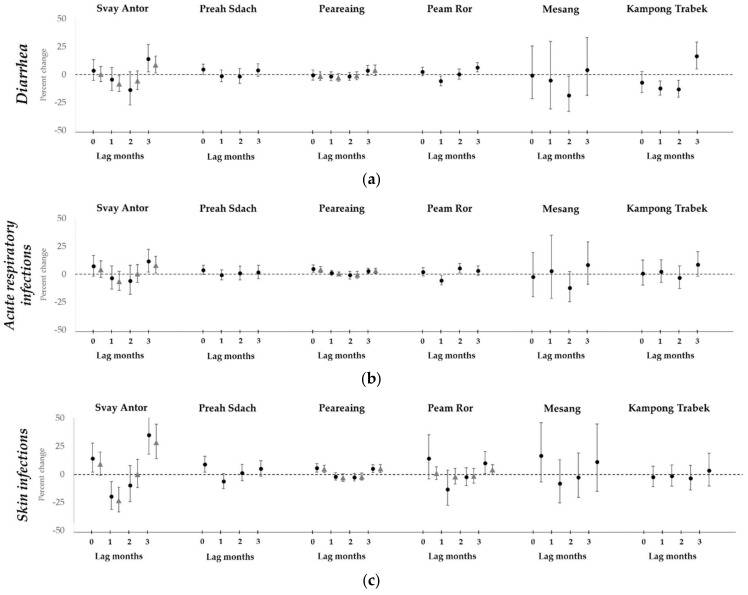
Percent change with 95% confidence interval in incidence rates of (**a**) diarrhea; (**b**) acute respiratory infections; and (**c**) skin infections, for every ten square kilometers of flood water in selected districts. Points marked with a circle are estimates using non-imputed data. Points marked with a triangle are estimates using imputed data.

At lag months 0 and 3, a trend across districts of positive associations between an increase in flood water and the incidence rate ratios (IRR) for diarrhea, acute respiratory infections, and skin infections (Figure 5) was observed; in other words, the number of visits for diarrhea, acute respiratory infections, and skin infections was affected by an increase in flood water one month and three months previously. There were moderate increases in the incidence rate of diarrhea of between 5% (95% confidence interval (CI): 1.00, 1.09) and 17% (95% CI: 1.05, 1.29) in four districts (Table S1), and in acute respiratory infections of 3% (95% CI: 1.01, 1.05) to 11% (95% CI: 1.02, 1.22). For skin infections, there were increases of 4% (95% CI: 1.00, 1.09) to 35% (95% CI: 1.18, 1.54). Additionally, the most substantial increases were seen in Kampong Trach district, which had increased incidence rates for diarrhea (IRR: 1.68, 95% CI: 1.40, 2.00 at lag 0 and IRR: 1.23, 95% CI: 1.00, 1.50 at lag 3), acute respiratory infections (IRR: 1.44, 95% CI: 1.28, 1.62), and skin infections (IRR: 3.86, 95% CI: 2.40, 6.21) at the same lag months.

Small declines in diarrhea incidence rates were seen at lag months 1 and 2, but not in all districts. The rates decreased by 6% (95% CI: 0.90, 0.99) to 26% (95% CI: 0.65, 0.90) in five districts, with the 26% decline seen in Kampong Trach district. There was no evidence for higher skin infections at lag months 1 and 2, but a pattern towards a decrease in incidence rates across districts was seen, with evidence for a decline in one district (IRR imputed data: 0.77, 95% CI: 0.67, 0.89, and IRR non-imputed data: 0.81, 95% CI: 0.69, 0.94). There was no evidence across the districts for a change in the incidence rate of acute respiratory infections, except for a decline at lag month 1 (IRR: 0.94, 95% CI: 0.90, 0.99) and an elevation at lag month 2 (IRR: 1.05, 95% CI: 1.01, 1.10) in one district.

**Figure 6:**
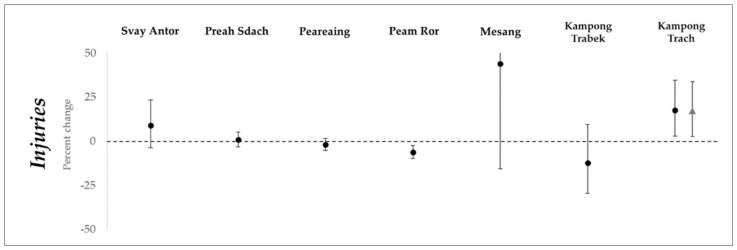
Percent change with 95% confidence interval in incidence rates of injuries for every ten square kilometers of flood water, in selected districts. Points marked with a circle are estimates using non-imputed data. Points marked with a triangle are estimates using imputed data.

For injuries, which were measured only at lag month 0, there was little evidence for variation in injuries, except an excess of injuries in one district (IRR: 1.17, 95% CI: 1.03, 1.34) and a small decline in a second district (IRR: 0.94, 95% CI: 0.90, 0.98) (Figure 6).

No associations between flood water and the incidence rate of normal and complicated deliveries at healthcare facilities were seen. Out of the 11 districts, declines in the incidence rate of outpatient consultations were observed at lag month 1 in Peam Ror (IRR: 0.95, 95% CI: 0.91, 0.99), and at lag month 1 (IRR: 0.90, 95% CI: 0.82, 0.99) and lag month 2 (IRR: 0.88, 5% CI: 0.79, 0.98) in Kampong Trach (results not shown). There were no other negative associations between outpatient consultations and flood water. The incidence rate of home deliveries increased (IRR: 1.39, 95% CI: 1.20, 1.60) in Kampong Trabek district, but did not change in the other districts.

The small number of visits reported at healthcare facilities and the minimal variation in flood water in Angkor Chey and Chhouk produced extreme and imprecise estimates and confidence intervals in the two districts and for vector-borne diseases and NCDs. The results are not presented here, but are available in Table S1.

## 4. Discussion

We observed a pattern of immediate increases in visits to healthcare facilities for respiratory infections, skin infections and diarrhea, followed by a decline, and a second increase three months later. There appeared to be no distinct relationship between floods and these health problems in the one to two months following floods. There was no sign that access to healthcare facilities declined during floods. However, the associations were small to moderate and few of our results were significant.

Our findings of a small effect during and three months after flooding were unexpected. Previous exposure to seasonal floods might have reduced the vulnerability of the population and explained the declines seen at lag months 1 and 2. If the public health system and population have already adapted to flooding, they are likely prepared to manage infections and diarrhea through control, prevention, and treatment measures that do not require facility-based care. During more severe floods, the provincial health departments have policies for community education programs in food hygiene and sanitation, for example, and can send mobile health units to affected villages. Consultations from mobile health units are reported to HMIS. Diarrhea has been linked to flooding before [19,20,21], but the strength of the relationship remains uncertain [22]. Similar to our results, another study from Cambodia indicated an association between flood events and diarrhea in only two of 16 provinces [23]. These results suggest a similar finding: diarrhea can increase after flooding, but the scale of the problem may be overestimated in flood-prone contexts.

Previous flood exposure may also explain why the rate of consultations and deliveries—our measures for access to healthcare—did not decline in most districts after floods. To our knowledge, results that show no change in access to healthcare have not been reported before in Cambodia. A study of health seeking behavior during floods to private providers, often the first point of care in Cambodia [24], would help to clarify these findings.

The size of the exposure appeared to be linked to the magnitude of the IRRs. The sharp spikes in flood water in Kampong Trach district during the 2011 and 2013 flood disasters were likely influencing the sizeable IRRs there, compared to the districts with more seasonal flood patterns. However, the low variability in flood water from districts with very little flooding was likely contributing to the wider, less precise estimates and intervals, for instance in Angkor Chey and Chhouk.

The HMIS data reflected a specific socioeconomic setting. In 2008, 34% of the Cambodian population lived under the poverty line, and wealth remained unequally distributed between rural and urban areas [24,25]. The first point of care for the poor was most often informal drug shops, but the next point of care was the public health sector [26]. In the poorer, rural areas, diarrheal disease and respiratory infections remained a problem, while the burden of disease in wealthier, urban areas had shifted towards injuries and NCDs [27]. The HMIS data represented the less economically-advantaged rural populations, and could partially explain the high number of cases, and significant results, seen for diarrhea and acute respiratory infections, including in the urban district of Svay Antor.

Displacement, poor sanitation, and contact with contaminated water were likely contributors to the increase in visits for acute respiratory infections, skin infections, and diarrhea that was observed three months after the onset of flooding. These indirect consequences of flooding have already been linked to acute respiratory infections, some types of skin infections, and diarrhea after disasters in other settings [20,28,29,30,31]. Flood water can damage the sewage system and contaminate the water supply, hindering basic hygiene and sanitation practices and increasing the risk of fecal-oral and water-borne disease transmission. Shelters that house the displaced can foster the transmission of infectious diseases. These are risk factors in Cambodia as well, where only 24% of the population use safely-managed water supplies [32] and 28,500 houses were recorded as damaged or destroyed by flooding between 1996 and 2013 [33]. A delay in seeking healthcare could also be contributing to the rise in visits, when the severity of the disease, transportation, and cost have all been identified as reasons for delaying care in Cambodia [34]. Regardless of the cause, the greatest health needs appear to occur in both the immediate and longer term. 

The average number of cases per month of vector-borne diseases and NCDs was surprisingly low. As a result, the analysis of the data produced very few precise estimates. Our estimate of seven cases of dengue fever per 100,000 people in Kampot, and 11 cases in Prey Veng, based on the HMIS data and the 2008 population, were much lower than expected. According to the Global Burden of Disease, the prevalence of dengue fever in Cambodia in 2008 was about 101 cases per 100,000 people [35], and a recent study of dengue in northern Cambodia found incidence rates of upwards of 1800 cases per 100,000 population [36]. It appeared that dengue cases were not captured in HMIS, perhaps because patients often sought care with private providers for dengue fever [24,37]. The low number of NCD cases was also surprising, since the estimated prevalence of diabetes in 2005 was between 5% and 11% [38], the self-reported prevalence of heart disease was 5% in 2007 [39], and NCDs represented 52% of adult deaths in 2010 [40]. However, between 2008 and 2013, data on heart disease and diabetes was only collected in HMIS from inpatient discharge diagnoses, so the cases in the HMIS dataset were those that required more advanced care. In addition, the majority of patients with NCDs in Cambodia were diagnosed and received care at private facilities [41,42]. Our analysis indicated little except that different approaches that include the private healthcare sector are needed to understand the relationship between vector-borne diseases, NCDs and floods in this setting.

There were several limitations to this study. Using HMIS data allowed us to systematically analyze the relationship between health and floods across the public health sector in multiple districts, but raised concerns about validity. The HMIS was facility-based data that did not include private providers, and the data was subject to selection bias through care-seeking behaviors. Because the data was monthly counts, the study was only able to show broad trends across time. In addition, the HMIS data used unstandardized diagnoses that can include a wide range of possible conditions (e.g., “skin infections” and “other injuries”), and changes in specific health conditions could not be identified. Nevertheless, the HMIS data had good internal consistency and completeness [15,16], and was a useful tool to compare health between districts. HMIS data also did not include information on whether the healthcare facilities themselves flooded, although the analysis of consultations and deliveries was used as an indicator of access to services.

Data were missing from the composite images due to cloud cover. The Global Flood Mapping project attempted to minimize cloud issues in its composite images [43], but it is probable that the true extent of flooding in the districts each month was underestimated, rather than overestimated. The wide confidence intervals and estimates seen in districts with the worst visibility on average may be due to the small amount of flood data visible during the low-visibility months. Despite the issues with cloud cover, the Global Flood Mapping project has successfully used the data to correctly detect flooding in Cambodia and elsewhere [14].

We defined flood water in our dataset as water that was detected outside the reference water boundaries. The reference water boundaries were from a static water map and did not show seasonal variations in water. Therefore, it was not possible to differentiate between normal seasonal flood water and more severe or unexpected floods. We presented flood water in square kilometers as a measure of the extent of the flooding, but this should not be interpreted as a reflection of severity.

The algorithm for identifying flood water in the three-day composite images required three days of observed water in a row. This method improved the true positive detection of water, reduced the misclassification of mountain shadow and inundated vegetation as water, and helped eliminate issues of missing data due to cloud dover [14], but water observed for less than three days was not identified as flood water. As a result, flash floods were not captured in this study. This method is still relevant in Cambodia, as inundation floods are more common and cause more damage than flash floods [8].

## 5. Conclusions

Our results indicate a link between floods and diarrhea, skin infections, and acute respiratory infections in the immediate and longer term in a flood-prone setting, but the effect was small. The effect might have been linked to previous exposure to flooding, although this could not be confirmed in our study. One potential for future research would be to use both community- and facility-based data to identify expected health risks after repeated flooding in flood-prone settings.

## Figures and Tables

**Table 1:** Healthcare facilities by district in Prey Veng and Kampot provinces, in 2010.

District	Hospitals	Health Centers (n)
**Prey Veng province**		
Kamchay Mear	Referral	11
Kampong Trabek	Referral	11
Mesang	Referral	10
Peam Ror	Referral	17
Peareaing	Referral	15
Preah Sdach	Referral	9
Svay Antor	Referral + provincial	16
**Kampot province**		
Angkor Chey	Referral	10
Chhouk	Referral	15
Kampong Trach	Referral	12
Kampot	Referral + provincial	11

**Table 2:** Summary of health and flood data between 2008 and 2013 by province and district.

District	Average Number of Visits, Per Month *						Mean Area Flooded Per Month (sq. Kilometers)	Mean Percent Cloud Cover Per Month
	Diarrhea	Acute Respiratory Infections	Skin Infections	Vector–Borne Diseases	Injuries	NCDs		
**Prey Veng province**	**730**	**2891**	**221**	**5**	**78**	**1**	**58.2**	**29.3**
Kamchay Mear	678	1818	134	7	74	0	5.2	37.2
Kampong Trabek	473	1344	113	3	26	0	22.0	28.9
Mesang	386	1848	192	1	42	0	10.5	31.5
Peam Ror	847	3525	258	5	110	1	106.0	22.4
Peareaing	1115	7280	347	10	120	6	90.4	27.0
Preah Sdach	1140	2758	384	4	152	0	105.8	28.0
Svay Antor	473	1666	121	3	23	2	67.2	30.3
**Kampot province**	**267**	**1050**	**116**	**57**	**171**	**1**	**8.5**	**32.6**
Angkor Chey	184	836	64	8	84	1	0.9	43.2
Chhouk	262	1190	182	118	175	1	0.5	31.7
Kampong Trach	191	916	130	16	140	0	16.2	33.6
Kampot	431	1 259	87	86	284	4	16.5	21.8
	Total number of visits, between 2008–2013							
	569,887	2,445,511	215,164	33,672	124,989	1549		

* Rounded to the nearest whole number. NCD: noncommunicable diseases.

## Supplementary Materials

The following are available online at http://www.mdpi.com/1660-4601/15/4/665/s1. Table S1: Incidence rate ratios and 95% confidence intervals for diarrhea, acute respiratory infections, vector-borne diseases, skin infections, noncommunicable diseases and injuries by district, corresponding to a ten square kilometer increase in flood water, controlled for season and year.
