# Supplementary material for: The Effect of Seasonal Floods on Health: Analysis of Six Years of National Health Data and Flood Maps
Source: Int J Environ Res Public Health. 2018 Apr 3;15(4):665. doi: 10.3390/ijerph15040665 (PMC5923707; doi:10.3390/ijerph15040665)
Supplement: Supplementary file 1 [file ijerph-15-00665-s001.pdf]

International Journal of
Environmental Research and Public Health

MDPI

Supplementary Materials

The Effect of Seasonal Floods on Health: Analysis of Six Years of National Health Data and Flood Maps

Dell D. Saulnier, Claudia Hanson, Por Ir, Helle Molsted Alvesson and Johan von Schreeb

Table S1. Incidence rate ratios and 95% confidence intervals for diarrhea, acute respiratory infections, vector-borne diseases, skin infections, noncommunicable diseases and injuries by district, corresponding to a ten square kilometer increase in flood water, controlled for season and year.

**Diarrhea**								
	Non-imputed data				*Imputed data*			
	Lag 0	Lag 1	Lag 2	Lag 3	Lag 0	Lag 1	Lag 2	Lag 3
Prey Veng								
Kamchay Mear	0.82 (0.49–1.35)	0.90 (0.51–1.57)	0.73 (0.52–1.01)	0.95 (0.52–1.72)				
Kampong Trabek	0.93 (0.84–1.03)	0.88 (0.82–0.94)	0.87 (0.80–0.95)	1.17 (1.05–1.29)				
Mesang	0.99 (0.78–1.25)	0.95 (0.69–1.30)	0.81 (0.67–0.99)	1.04 (0.81–1.33)				
Peam Ror	1.03 (0.99–1.07)	0.94 (0.90–0.99)	1.00 (0.96–1.05)	1.06 (1.02–1.11)				
Peareaing	0.99 (0.95–1.04)	0.98 (0.95–1.02)	0.98 (0.95–1.02)	1.04 (0.99–1.08)	0.99 (0.95–1.03)	0.97 (0.94–1.01)	0.99 (0.95–1.03)	1.04 (0.99–1.08)
Preah Sdach	1.05 (1.00–1.09)	0.99 (0.94–1.04)	0.98 (0.92–1.05)	1.04 (0.98–1.10)				
Svay Antor	1.04 (0.95–1.13)	0.96 (0.86–1.06)	0.86 (0.73–1.03)	1.14 (1.02–1.27)	1.00 (0.94–1.07)	0.92 (0.85–0.99)	0.95 (0.87–1.03)	1.09 (1.01–1.16)
Kampot								
Angkor Chey	0.03 (0.00–31.52)	0.83 (0.00–104.51)	0.00 (0.00–3.29)	4.01 (0.03–535.26)	2.14 (0.27–16.80)	2.75 (0.55–13.74)	0.22 (0.01–5.05)	22.54 (4.49–113.21)
Chhouk	0.00 (0.00–0.67)	2.2e+9 (0.00–1.6e+24)	0.00 (0.00–2.8e+7)	214.10 (0.00–5.6e+15)	0.00 (0.00–19.05)	297.45 (0.00–3.7e+12)	0.03 (0.00–3.5e+9)	163179.1 (0.00–5.3e+15)
Kampong Trach	1.68 (1.40–2.00)	0.76 (0.65–0.90)	0.74 (0.56–0.97)	1.16 (0.87–1.54)	1.55 (1.33–1.80)	0.79 (0.71–0.89)	0.85 (0.72–1.00)	1.23 (1.00–1.50)
Kampot	1.62 (0.22–11.66)	0.43 (0.08–2.21)	9.62 (0.80–115.87)	1.30 (0.40–4.25)	0.49 (0.11–2.04)	0.96 (0.31–2.97)	1.48 (0.50–4.42)	1.32 (0.46–3.79)
Acute respiratory infections								
	Non-imputed data				*Imputed data*			
	Lag 0	Lag 1	Lag 2	Lag 3	Lag 0	Lag 1	Lag 2	Lag 3
Prey Veng								
Kamchay Mear	0.61 (0.34–1.09)	0.74 (0.48–1.14)	0.98 (0.69–1.40)	1.21 (0.92–1.59)				
Kampong Trabek	1.01 (0.90–1.12)	1.02 (0.93–1.13)	0.97 (0.87–1.07)	1.08 (0.98–1.20)				
Mesang	0.97 (0.80–1.19)	1.03 (0.79–1.34)	0.88 (0.75–1.02)	1.08 (0.91–1.28)				

Peam Ror	1.02 (0.98–1.06)	0.94 (0.90–0.99)	1.05 (1.01–1.10)	1.03 (0.99–1.07)				
Peareaing	1.05 (1.02–1.08)	1.01 (0.99–1.03)	0.99 (0.96–1.02)	1.03 (1.00–1.05)	1.04 (1.01–1.07)	1.00 (0.99–1.02)	0.99 (0.97–1.02)	1.03 (1.01–1.05)
Preah Sdach	1.04 (0.99–1.08)	0.99 (0.95–1.04)	1.01 (0.95–1.07)	1.02 (0.96–1.08)				
Svay Antor	1.07 (0.98–1.07)	0.96 (0.87–1.07)	0.94 (0.82–1.08)	1.11 (1.02–1.22)	1.04 (0.98–1.12)	0.94 (0.86–1.02)	1.00 (0.93–1.08)	1.08 (1.01–1.16)
Kampot								
Angkor Chey	2.98 (0.32–28.15)	8.02 (2.18–29.46)	0.49 (0.00–248.27)	1.72 (0.02–168.71)				
Chhouk	11167.12 (0.00–1.8e+12)	48187.75 (0.00–2.2e+13)	0.00 (0.00–1069161)	0.00 (0.00–290249.7)				
Kampong Trach	1.44 (1.28–1.62)	0.92 (0.83–1.01)	0.89 (0.76–1.03)	1.20 (1.05–1.38)				
Kampot	0.84 (0.30–2.33)	0.85 (0.30–2.39)	1.10 (0.37–3.32)	0.40 (0.16–0.96)				
Vector-borne diseases								
	Non-imputed data				*Imputed data*			
	Lag 0	Lag 1	Lag 2	Lag 3	Lag 0	Lag 1	Lag 2	Lag 3
Prey Veng								
Kamchay Mear	0.36 (0.02–5.81)	0.24 (0.01–8.78)	0.47 (0.03–7.91)	1.68 (0.04–70.87)				
Kampong Trabek	1.28 (0.75–2.16)	0.96 (0.53–1.74)	0.60 (0.25–1.43)	0.63 (0.25–1.56)				
Mesang	1.56 (0.29–8.27)	2.31 (0.25–21.07)	2.72 (0.39–19.01)	1.46 (0.23–9.43)				
Peam Ror	1.25 (0.92–1.70)	0.99 (0.67–1.48)	0.64 (0.29–1.41)	1.30 (0.60–2.81)				
Peareaing	0.77 (0.60–0.99)	0.75 (0.58–0.97)	1.02 (0.71–1.47)	1.03 (0.77–1.37)				
Preah Sdach	0.99 (0.74–1.33)	0.96 (0.67–1.38)	1.05 (0.76–1.45)	1.00 (0.74–1.37)				
Svay Antor	2.07 (1.39–3.10)	0.75 (0.46–1.21)	0.79 (0.46–1.34)	0.96 (0.60–1.55)				
Kampot								
Angkor Chey	0.00 (0.00–94.71)	0.00 (0.00–3.65)	0.00 (0.00–0.37)	0.00 (0.00–2.24)	0.01 (0.00–142.57)	0.02 (0.00–2.37)	0.00 (0.00–1.25)	0.00 (0.00–1.62)
Chhouk	7.8e+23 (3.0e+8–2.0e+39)	12.87 (0.00–9.1e+15)	6.9e+40 (2.3e+15–2.0e+66)	0.00 (0.00–0.00)	2.0e+9 (0.12–3.5e+18)	582.59 (0.00–1.2e+14)	0.02 (0.00–1.0e+12)	0.00 (0.00–50.79)
Kampong Trach	0.83 (0.28–2.47)	0.27 (0.09–0.85)	2.26 (0.95–5.42)	0.92 (0.45–1.86)	0.48 (0.26–0.88)	0.30 (0.14–0.61)	2.35 (1.28–4.34)	0.91 (0.56–1.47)
Kampot	0.35 (0.09–1.41)	0.98 (0.17–5.71)	0.23 (0.04–1.20)	0.74 (0.18–3.01)	0.29 (0.07–1.29)	0.90 (0.15–5.35)	0.18 (0.04–0.96)	1.20 (0.40–3.65)
Skin infections								
	Non-imputed data				*Imputed data*			
	Lag 0	Lag 1	Lag 2	Lag 3	Lag 0	Lag 1	Lag 2	Lag 3
Prey Veng								
Kamchay Mear	0.81 (0.53–1.23)	0.91 (0.54–1.55)	0.85 (0.55–1.30)	0.81 (0.51–1.29)				
Kampong Trabek	0.98 (0.89–1.07)	0.99 (0.90–1.09)	0.97 (0.86–1.08)	1.04 (0.90–1.19)				
Mesang	1.17 (0.94–1.46)	0.92 (0.75–1.13)	0.98 (0.80–1.19)	1.11 (0.85–1.45)				
Peam Ror	1.14 (0.96–1.35)	0.87 (0.73–1.04)	0.98 (0.90–1.06)	1.10 (1.01–1.20)	1.01 (0.96–1.07)	0.98 (0.92–1.06)	0.99 (0.92–1.05)	1.04 (1.00–1.09)
Peareaing	1.06 (1.02–1.10)	0.98 (0.95–1.02)	0.98 (0.94–1.01)	1.05 (1.02–1.09)	1.05 (1.02–1.08)	0.97 (0.94–1.01)	0.98 (0.95–1.02)	1.05 (1.02–1.09)
Preah Sdach	1.09 (1.02–1.16)	0.94 (0.88–1.01)	1.02 (0.95–1.09)	1.05 (0.99–1.12)				

Svay Antor	1.14 (1.02–1.28)	0.81 (0.69–0.94)	0.91 (0.76–1.08)	1.35 (1.18–1.54)	1.09 (0.99–1.20)	0.77 (0.67–0.89)	1.00 (0.89–1.13)	1.28 (1.14–1.44)
Kampot								
Angkor Chey	1.33 (0.22–7.99)	0.81 (0.04–15.46)	0.04 (0.00–16.41)	0.14 (0.00–58.17)				
Chhouk	1.14 (0.00–1.7e+9)	1926.42 (0.00–2.3e+13)	9.4e+10 (0.00–6.9e+23)	0.00 (0.00–6.9e+8)				
Kampong Trach	3.86 (2.40–6.21)	1.04 (0.91–1.19)	0.44 (0.17–1.14)	0.94 (0.45–1.96)	2.82 (2.41–3.29)	1.07 (0.98–1.17)	0.74 (0.48–1.15)	1.22 (0.81–1.85)
Kampot	0.48 (0.16–1.41)	0.94 (0.38–2.30)	1.75 (0.50–6.15)	0.79 (0.31–1.99)				
Noncommunicable diseases								
	Non-imputed data				*Imputed data*			
	Lag 0	Lag 1	Lag 2	Lag 3	Lag 0	Lag 1	Lag 2	Lag 3
Prey Veng								
Kamchay Mear	0.00 (0.00–13.62)	5.97 (0.00–111891.5)	13.61 (0.02–11675.79)	0.00 (0.00–143726.6)				
Kampong Trabek	0.69 (0.05–9.10)	0.45 (0.00–23.06)	2.65 (0.53–13.17)	0.32 (0.05–2.13)				
Mesang	0.11 (0.01–0.98)	2.08 (0.12–36.93)	0.66 (0.06–6.97)	0.30 (0.04–2.04)				
Peam Ror	0.74 (0.38–1.46)	1.10 (0.59–2.04)	1.36 (0.57–3.28)	0.80 (0.46–1.37)				
Peareaing	1.02 (0.83–1.26)	1.13 (0.87–1.48)	1.00 (0.76–1.32)	1.57 (1.21–2.03)	1.02 (0.92–1.13)	1.03 (0.86–1.22)	1.05 (0.93–1.18)	0.94 (0.82–1.08)
Preah Sdach	1.89 (0.30–11.87)	0.06 (0.00–51410.43)	0.00 (0.00–307.59)	4.0e+12 (0.00–1.9e+34)				
Svay Antor	1.05 (0.66–1.67)	0.62 (0.33–1.18)	0.90 (0.50–1.62)	1.40 (0.97–2.03)				
Kampot								
Angkor Chey	0.00 (0.00–1869.58)	0.73 (0.00–1165.72)	15469.45 (0.04–5.5e+10)	7.07 (0.00–45910.69)				
Chhouk	0.00 (0.00–0.00)	5.0e+122 (2.1e+96–1.1e+150)	1.0e+60 (0.00–2.5e+125)	0.00 (0.00–0.00)	0.00 (0.00–9.3e+44)	1.9e+35 (1.0e+10–3.6e+78)	4.0e+16 (0.00–2.7e+79)	0.00 (0.00–3.9e+13)
Kampong Trach	3.5e+50 (1.0e+50–1.2e+51)	0.00 (0.00–0.00)	2.0e+94 (2.7e+92–1.6e+94)	0.00 (0.00–0.00)				
Kampot	0.16 (0.00–24.31)	14.04 (0.02–9471.05)	0.00 (0.00–46.35)	572.7 (0.28–1171946)	0.12 (0.01–1.80)	0.27 (0.01–5.48)	3.65 (0.04–326.34)	0.86 (0.03–21.89)
Injuries								
	Non-imputed data				*Imputed data*			
	Lag 0	Lag 1	Lag 2	Lag 3	Lag 0	Lag 1	Lag 2	Lag 3
Prey Veng								
Kamchay Mear	0.48 (0.09–2.46)							
Kampong Trabek	0.88 (0.70–1.10)							
Mesang	1.44 (0.85–2.46)							
Peam Ror	0.94 (0.90–0.98)							
Peareaing	0.98 (0.95–1.02)							

Preah Sdach	1.01 (0.97–1.05)	
Svay Antor	1.09 (0.96–1.24)	
Kampot		
Angkor Chey	9.06 (2.18–37.62)	
Chhouk	0.00 (0.00–2.29)	
Kampong Trach	1.17 (1.09–1.62)	1.17 (1.03–1.34)
Kampot	0.91 (0.51–1.61)	

Rate ratios and confidence intervals written in bold, red text are statistically significant. Estimates are rounded to two digits.
